# Enzyme-constrained genome-scale modeling resolves growth-production trade-offs in fermentative biohydrogen production

**DOI:** 10.1016/j.ese.2026.100706

**Published:** 2026-05-14

**Authors:** Wei Xing, Jianfeng Liu, Bin Liu, Yanan Hou, Jia Zhang, Shuang Gao, Ai-Jie Wang, Qianqian Yuan, Nan-Qi Ren, Cong Huang

**Affiliations:** a National Technology Innovation Center of Synthetic Biology, Tianjin Institute of Industrial Biotechnology, Chinese Academy of Sciences, Tianjin, 300308, China; b Key Laboratory of Environmental Biotechnology, Research Center for Eco-Environmental Sciences, Chinese Academy of Sciences, Beijing, 100085, China; c Tianjin Key Laboratory of Aquatic Science and Technology, School of Environmental and Municipal Engineering, Tianjin Chengjian University, Tianjin, 300384, China; d State Key Laboratory of Urban Water Resource and Environment, Harbin Institute of Technology (Shenzhen), Shenzhen, 518055, China

**Keywords:** Anaerobic biohydrogen production, Enzyme-constrained genome-scale metabolic network model, Hydrogen production mechanism

## Abstract

Hydrogen is central to sustainable energy systems, with biological production from waste offering a low-energy, environmentally compatible route. Anaerobic dark fermentation by microbes converts organic substrates into hydrogen, yet yields remain limited by competing metabolic pathways and poor understanding of cellular resource allocation in hydrogen-producing strains. Conventional genome-scale models rely on stoichiometric constraints alone, often failing to capture realistic enzyme limitations or strain-specific biomass composition. Here we show that an enzyme-constrained genome-scale metabolic model (ecGEM) of the hydrogen-producing bacterium *Ethanoligenens harbinense* YUAN-3, built with experimentally measured biomass composition and predicted *k*_cat_ values, quantitatively captures the trade-off between growth and hydrogen production. Enzyme constraints eliminate unrealistic flux predictions of standard models, accurately matching experimental growth rates and yields, and reveal that diversion of carbon and NADH flux into glutamate and glutamine biosynthesis enhances hydrogen production by reducing ethanol formation. In silico single-gene knockouts identify targets such as phosphoglycerate kinase that increase hydrogen flux by up to 30% under low-carbon conditions. These findings elucidate system-level metabolic regulation in fermentative hydrogen production and provide a predictive framework for rational strain engineering. The approach offers a scalable platform for optimizing biohydrogen processes and advancing sustainable hydrogen economies.

## Highlights

• Enzyme-constrained genome-scale models accurately predict growth–hydrogen trade-offs in fermentative bacteria.
• Amino acid biosynthesis diverts NADH flux to enhance hydrogen yields in the model strain YUAN-3.
• In silico gene knockouts identify targets increasing hydrogen production by up to 30%.
• The ecGEM framework advances rational metabolic engineering for sustainable biohydrogen technologies.

## Graphical abstract

**Figure:**
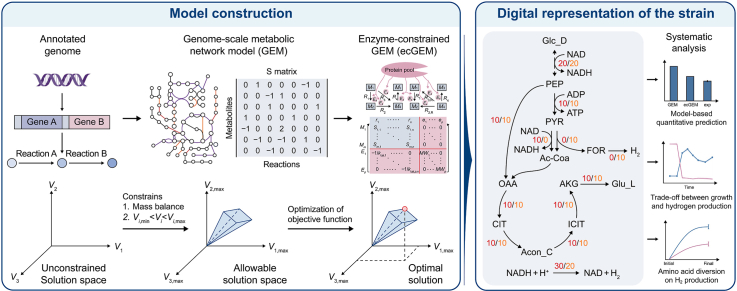

## 1 Introduction

Hydrogen is an essential component in sustainable energy systems [1]. Currently, most of the world's hydrogen is produced from fossil fuels, which depletes non-renewable resources and exacerbates the greenhouse effect [2]. In contrast, anaerobic biohydrogen production presents distinct advantages, including broad substrate availability, low energy consumption, and strong environmental compatibility [3]. Moreover, it achieves waste-resource utilization during hydrogen production. Therefore, it is widely regarded as one of the most commercially promising routes for biological hydrogen production.

As a result, extensive research has been conducted to improve the efficiency of anaerobic biohydrogen production. On the one hand, researchers have improved hydrogen yields by adjusting external conditions such as pH, temperature, and medium composition [4,5]. On the other hand, metabolic engineering strategies, including the knockout or attenuation of competing pathways, have been employed to enhance hydrogen production efficiency [6]. Nevertheless, most existing research relies on empirical methods, and investigations of hydrogen-producing pathways are often conducted in isolation from the strains' overall metabolic networks. However, there remains a limited systematic understanding of metabolic regulation in hydrogen-producing strains.

The genome-scale metabolic network models (GEMs), cell models that mathematically represent cellular biochemical networks, have become pivotal tools in systems biology [[7], [8], [9]]. GEMs demonstrate remarkable value across fields such as fundamental biology, microbial engineering, medicine, and environmental biotechnology [[10], [11], [12], [13]]. Notably, existing GEM studies on anaerobic biohydrogen production are typically constrained by stoichiometric assumptions and often lack strain-specific biomass composition data, limiting their ability to analyze metabolic allocation in such strains. Recently, with enzyme turnover numbers (*k*_cat_) introduced as additional constraints, enzyme-constrained genome-scale metabolic models (ecGEMs) can account for both metabolic reaction fluxes and cellular enzyme resource allocation at the level of the metabolic network, thereby yielding more biologically feasible flux distributions [14,15]. In this study, we used an ecGEM to systematically analyze the metabolic trade-offs among growth, by-product formation, and hydrogen generation during dark fermentation, and to identify key metabolic processes that limit hydrogen yield and their potential regulatory mechanisms.

We selected the hydrogen-producing strain *Ethanoligenens harbinense* YUAN-3 (YUAN-3) and constructed an ecGEM for this strain. Compared with existing hydrogen-producing GEM studies, this study integrates the experimentally measured biomass composition of strain YUAN-3 into the model construction process. Likewise, it incorporates DLkcat-predicted *k*_cat_ values to enforce enzymatic kinetic constraints and manually refines the model based on the strain's carbon-source utilization and metabolic characteristics. Additionally, this model quantitatively predicts growth rate and hydrogen yield, systematically elucidating the resource allocation strategies underlying hydrogen generation. Moreover, it revealed the key role of amino acid biosynthesis in hydrogen metabolism. To further explore engineering opportunities, we performed in silico single-gene perturbation analyses to prioritize candidate genetic targets within hydrogen-producing pathways. Collectively, this study provides a theoretical foundation for the mechanistic understanding and performance optimization of fermentative hydrogen production. It also offers a modeling framework that supports its eventual industrial application.

## 2 Materials and methods

### 2.1 Flux balance analysis

Flux balance analysis (FBA) is a computational approach based on GEMs that assumes a metabolic steady state to predict intracellular flux distributions [16]. Within this framework, upper and lower bounds are assigned to each reaction, such as substrate uptake rates, reaction reversibility, and environmental conditions. A corresponding linear programming problem is then formulated, with an objective function that maximizes either the specific growth rate or the yield of a target product. All FBA calculations were performed using the COBRApy software package [17].

### 2.2 Model construction and preliminary refinement

The genome of strain YUAN-3 was sequenced, and the assembled genome has been deposited under the accession number GCF_003020045.1. The genome file was uploaded to the RAST system for annotation (https://rast.nmpdr.org/) [18]. Open reading frames were automatically identified and functionally assigned based on the subsystem classification approach. The annotation job was submitted after entering the required biological and parameter information. Upon completion, the annotated results from RAST were imported into ModelSEED to semi-automatically generate a draft GEM [17]. The draft GEM was then manually curated in accordance with established protocols for metabolic model reconstruction, including specifying the growth medium composition and formulating the biomass equation [19]. The final curated model has been publicly released in Systems Biology Markup Language format to facilitate reuse and reproducibility, and its memote score details are provided in Supplementary Fig. S1. This model adopts a compartmental structure comprising a cytosolic and an extracellular compartment [19]. All intracellular metabolic reactions were assigned to the cytosol, while nutrient uptake and product secretion were represented through exchange and transport reactions linking the cytosolic and extracellular compartments. The extracellular compartment was treated as a well-mixed reservoir with fixed boundary conditions. The check_mass_balance function in COBRApy verified the elemental and charge balances of all reactions within the model. Excluding exchange reactions, a total of 42 reactions were identified as mass- or charge-imbalanced. These reactions were manually inspected by cross-referencing the Kyoto Encyclopedia of Genes and Genomes (KEGG) and the Genetic and Genomic knowledgebase databases, and the chemical formulas and charge states of the associated metabolites were corrected as needed. Manual curation resolved 18 imbalanced reactions, thereby improving the overall stoichiometric consistency of the model. The remaining imbalanced reactions primarily correspond to macromolecular composition reactions, which are standard components of genome-scale metabolic reconstructions. However, these reactions do not affect intracellular stoichiometric consistency.

### 2.3 Biomass equation reconstruction

The composition of the biomass equation is given by equation (1), where *R*_*i*_ and *B*_*j*_ note precursor and biomass-component metabolites; *i* and *j* index these metabolites; *S*_*i*_ and *S*_*j*_ are the corresponding stoichiometric coefficients; and *X* denotes the biomass pseudo-metabolite. The reaction was normalized to 1 g dry weight (DW) by equation (2), where *M*_*i*_ and *M*_*j*_ denote the molecular weights of *R*_*i*_ and *B*_*j*_, respectively.

(1) $$\sum_{i}S_{i}R_{i}=X+\sum_{j}S_{j}B_{j}$$

(2) $$\frac{\sum_{i}S_{i}M_{i}-\sum_{j}S_{j}M_{j}}{1000}=1$$

To accurately construct the biomass equation, we measured the contents of proteins, nucleic acids, and glycogen, as well as their corresponding small molecules. We used the Tris-phenol method for protein extraction, followed by centrifugation, precipitation, and washing to isolate the protein. Protein concentration was determined using the Bradford method at 595 nm. The method used for amino acid determination is detailed in Section 2.7. DNA and RNA were extracted using kits, and their concentrations and purities were measured using an ultraviolet spectrophotometer (Nanodrop 2000, Thermo, USA). The nucleotide ratios of DNA and RNA were calculated using FASTA files containing the coding sequences. For glycogen content measurement, 50 mg of dry biomass was sonicated and centrifuged; the supernatant was analyzed using the phenol-sulfuric acid colorimetric method, with absorbance measured at 490 nm and content calculated from a standard curve. The ratios of cell wall substances, lipopolysaccharides, lipids, inorganic ions, and soluble pool components were referenced from the composition data of *Escherichia coli i*AF1260. All component results were used to recalculate the stoichiometric coefficients in the biomass equation. Further details of the biomass composition measurements and the recalculated stoichiometric coefficients are provided in Supplementary Table S1.

We incorporated growth-associated maintenance (GAM) and non-growth-associated maintenance (NGAM) to represent cellular energy requirements. Both parameters were adopted from a previously reported metabolic model: GAM was set to 26.48 mmol adenosine triphosphate (ATP) per g DW, and NGAM was set to 0.81 mmol ATP per g DW [20].

### 2.4 Gap-filling metabolic networks for biomass

During model curation, a predicted growth rate of zero indicated that the reconstructed metabolic network could not support the synthesis of one or more biomass precursors, typically due to missing reactions resulting from incomplete genome annotation. We therefore applied a targeted gap-filling procedure focused exclusively on the biosynthesis of the biomass precursor.

Specifically, we used COBRApy to iteratively test the producibility of individual biomass components. When a precursor metabolite could not be synthesized, FBA was applied to trace upstream metabolic bottlenecks. Missing reactions were then identified and supplemented based on combined evidence from the KEGG database and genome annotation. This procedure was repeated until all biomass precursors became producible.

Through targeted gap-filling, we added 109 reactions, including transport reactions, to the model and refined the associated metabolite annotations. The reversibility of the newly added reactions was determined using standard Gibbs free energy estimates from the eQuilibrator database. After curation, the model sustained non-zero growth under defined conditions, indicating that all biomass precursor pathways were functionally connected. The complete list of gap-filled reactions, including reaction identifiers and functional annotations, is provided in Supplementary Table S2.

### 2.5 Strain cultivation

The hydrogen-producing strain YUAN-3, isolated previously, was used in this study [21]. For the experiments in this study, the strain was cultured in 100 mL anaerobic serum bottles containing 70 mL of anaerobic medium at 37 °C and 220 rpm for 12–24 h. The culture was successively transferred three times to ensure strain stability. The anaerobic medium contained 1.5 g L^−1^ KH_2_PO_4_, 4 g L^−1^ NaCl, 0.5 g L^−1^ L-cysteine, 5 g L^−1^ beef extract, 0.1 g L^−1^ MgCl_2_·6H_2_O, 0.1 g L^−1^ FeSO_4_·7H_2_O, 1 mL L^−1^ vitamin solution, 1 mL L^−1^ trace element solution, 10 g L^−1^ glucose, 5 g L^−1^ tryptone, and 5 g L^−1^ yeast extract. The pH of the medium was adjusted to 6.8 using NaOH and HCl. Bottles were flushed with high-purity nitrogen gas to remove residual oxygen and maintain anaerobic conditions. To assess carbon source utilization, glucose was individually replaced with each carbon source in the culture medium, and all experiments were performed in triplicate. Before inoculation, cells were washed with phosphate-buffered saline to remove residual medium components and minimize carbon carryover. Cultures were incubated for 72 h, and growth was considered positive when the biomass concentration reached or exceeded 0.3 g DW L^−1^. A carbon source was classified as growth-supporting if at least two out of three parallel cultures met this criterion.

### 2.6 Product analysis

Fermentation broth samples were collected inside an anaerobic workstation using a 2 mL syringe. These samples were then filtered through 0.22 μm membrane filters to remove impurities before liquid-phase analysis. Gas samples were taken using a 50 mL gas-tight syringe directly from the anaerobic bottles, and gas volumes were recorded for analysis (Supplementary Text S1). All measurements were conducted using three independent biological replicates.

Glucose and the major soluble metabolites in the fermentation broth were quantified using a high-performance liquid chromatography system (Agilent 1260, Agilent, USA). Separation was performed on an Aminex HPX-87H carbohydrate analysis column (300 mm × 7.8 mm, Bio-Rad, USA) maintained at 55 °C. Detection was performed with a refractive index detector set at 50 °C. The mobile phase consisted of 5 mmol L^−1^ H_2_SO_4_, delivered at a flow rate of 0.6 mL min^−1^ under isocratic conditions.

Hydrogen concentration was determined using a gas chromatograph (GC; Agilent7890B, Agilent, USA) [22]. The injector, column oven, and detector were maintained at 100, 100, and 120 °C, respectively. High-purity argon was used as the carrier gas at a flow rate of 40 mL min^−1^.

### 2.7 Amino acid content determination

During fermentation, broth samples were collected for amino acid analysis. Amino acid measurements were performed in biological triplicate for each experimental condition. The amino acid concentrations measured represent the free amino acids present in the fermentation broth. The collected samples were filtered through a 0.22 μm membrane filter to remove microbial cells and suspended particles, and the resulting filtrates were used for subsequent amino acid determination.

The amino acid composition and concentration in the extracted samples were determined using an automatic amino acid analyzer. Quantification was performed using the external standard method. We employed an ion-exchange separation column (4.6 mm inner diameter × 60 mm length; protein hydrolysate column) and a reaction column (4.6 mm inner diameter × 40 mm length; packed with inert carborundum particles). During the analysis, the separation column was maintained at 57 °C, while the reaction column was kept at 135 °C. Detection wavelengths were set at 570 and 440 nm, and the sample injection volume was 20 μL.

### 2.8 Enzyme-constrained model construction

An ecGEM was developed from the constructed GEM by integrating *k*_cat_. The GEM was first preprocessed, and all gene identifiers were mapped to UniProt identifiers to retrieve protein sequences and subunit numbers [23]. Additionally, 44 genes were newly added to the model to improve GPR coverage, and the resulting information was subsequently used to calculate enzyme molecular weights (Supplementary Table S1). Enzyme mass fractions were estimated by combining the experimentally measured total cellular protein content with relative gene expression levels from publicly available RNA-seq data; these transcriptomic data were used solely as allocation weights to distribute the global enzyme pool, rather than as direct measures of absolute protein abundance. *k*_cat_ values were primarily obtained from DLkcat predictions [24], resulting in a total of 1006 reactions with matched enzyme kinetic parameters (Supplementary Table S4). These *k*_cat_ values were then integrated into the model using ECMpy [25], and further calibrated. Detailed procedures and computational settings are provided in Supplementary Texts S2–S6.

## 3 Results and discussion

### 3.1 GEM construction and optimization

**Fig. 1:**
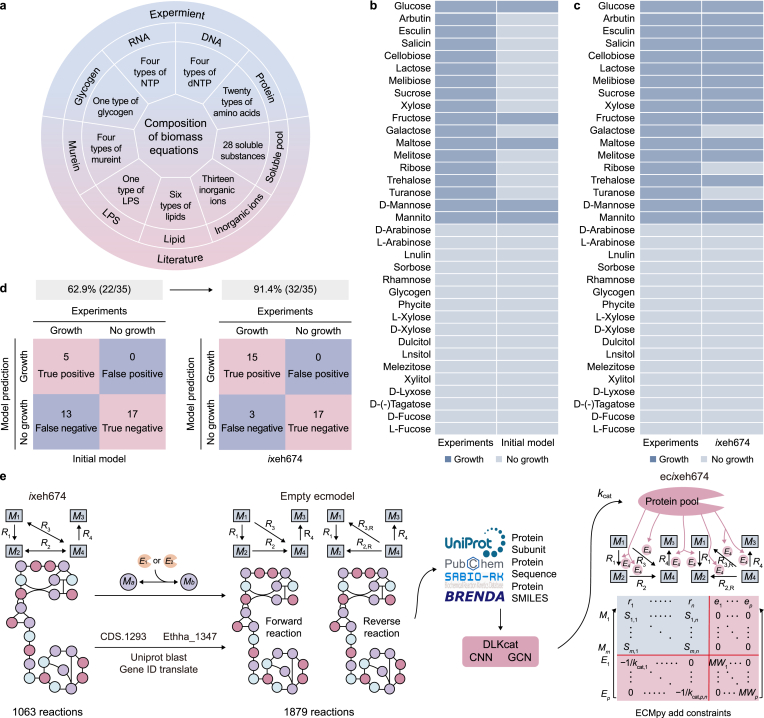
**Correction of the genome-scale metabolic network model (GEM) and construction of the enzyme-constrained genome-scale metabolic network model (ecGEM). a**, Composition of precursor substances in the biomass equation. NTP, nucleoside triphosphates; dNTP, deoxyribonucleoside triphosphates; LPS, lipopolysaccharides. **b**–**c**, Comparison of experimental and simulated growth phenotypes for 35 carbon sources before (**b**) and after (**c**) correction. **d**, Comparison of simulated and experimental results using different substrates before and after model correction. The percentages indicate the overall prediction accuracy, calculated as the proportion of correctly predicted phenotypes among the 35 tested carbon sources. Pink box: consistent results; blue box: inconsistent results. **e**, Process of obtaining enzyme turnover numbers (*k*_cat_) and constructing the ecGEM. *M*_1_–*M*_*m*_ denote metabolites, *R*_1_–*R*_*n*_ denote reactions, *E*_*1*_–*E*_*p*_ denote enzymes, *r*_*1*_–*r*_*n*_ denote reactions in the model, *e*_1_–*e*_*p*_ denote enzyme usages, *MW*_1_–*MW*_*p*_ denote the molecular weights of enzymes, and *S*_*m,n*_ is the stoichiometric coefficient of metabolite *m* in reaction *n*. Subunit denotes enzyme subunit number or composition, Sequence denotes the protein amino acid sequence, and SMILES denotes the simplified molecular input line entry system. CNN, convolutional neural network; GCN, graph convolutional network.

A high-quality GEM is an essential tool for quantitative cellular analysis and plays a key role in understanding and optimizing cellular metabolism [26]. We constructed the first GEM for YUAN-3, designated *i*xeh674, comprising 674 genes, 977 metabolites, and 1063 reactions. We also reconstructed a strain biomass equation for *i*xeh674 by integrating experimentally measured data on protein, DNA, RNA, glycogen, and amino acid composition with the *i*AF1260 model (Fig. 1a) [27]. Compared with the original biomass equation, the reconstructed equation substantially improved both the accuracy of growth rate predictions and the reliability of flux distribution.

Model performance was validated by simulating the strain's ability to utilize 35 individual carbon sources using FBA. The initial model achieved a qualitative prediction accuracy of 64.71% (Fig. 1b). Based on KEGG and MetaCyc pathways, degradation pathways for substrates that showed discrepancies between simulated and experimental data were manually corrected. All gene identifiers in the model were converted to UniProt identifiers. Reactions lacking gene annotations were subsequently cross-annotated and revised using the deep-learning-based tool ECRECer [28], resulting in 44 newly annotated genes (Supplementary Table S1). This process yielded the final GEM, *i*xeh674. After correction, the overall prediction accuracy improved to 91.42% (Fig. 1c). The final model correctly identified 15 true positives (substrates that can be used in both in vivo experiments and simulations) and 17 true negatives (substrates unsuitable for both experiments and simulations) (Fig. 1d). These results indicate that the GEM's core metabolic and transport functions were significantly enhanced, providing a solid foundation for subsequent phenotype prediction and mechanistic understanding of the strain.

Because the GEM accounts only for stoichiometric constraints, enzyme resource parameters reflecting protein limitations were incorporated to further improve model accuracy. The *k*_cat_ values for YUAN-3 were obtained from DLkcat and incorporated into *i*xeh674 using ECMpy to construct ec*i*xeh674 (Fig. 1e).

### 3.2 Integrative analysis of growth and hydrogen production in YUAN-3

**Fig. 2:**
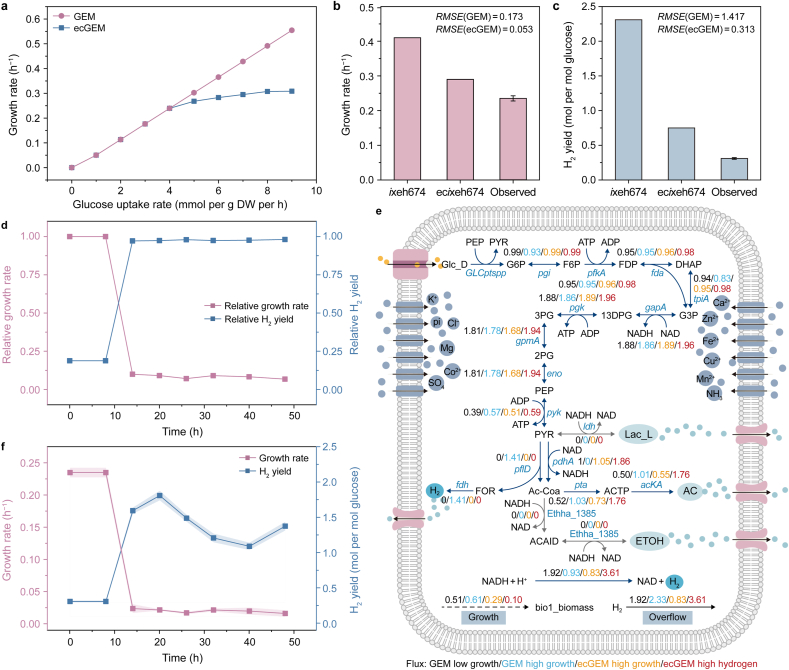
**Simulation predicting growth and hydrogen-production process of YUAN-3 by *i*xeh674 and ec*i*xeh674. a**, Growth rates simulated under different carbon uptake rates using *i*xeh674 and ec*i*xeh674. DW: dry weight. **b**–**c**, Comparison of predictions for growth rate (**b**) and hydrogen yield (**c**) from *i*xeh674 and ec*i*xeh674 with experimental results. **d**, Trade-off relationship between strain growth rate and hydrogen yield. **e**, Metabolic flux simulations for the strain under different states by *i*xeh674 and ec*i*xeh674. Black arrows indicate material transport or exchange reactions across the cell boundary, blue arrows indicate the hydrogen-production pathway, and gray arrows indicate competing pathways for hydrogen production. Flux values are colored according to condition: black, *i*xeh674 flux distribution at low growth rate; blue, *i*xeh674 at optimal growth rate; yellow, ec*i*xeh674 at optimal growth rate; red, ec*i*xeh674 during high hydrogen yield. **f**, Experimentally observed variations in growth rate and hydrogen yield. The shaded areas represent the standard deviations of parallel samples. Glc_D, D-glucose; G6P, D-glucose-6-phosphate; F6P, D-fructose-6-phosphate; FDP, D-fructose-1,6-bisphosphate; DHAP, glycerone-phosphate; G3P, glyceraldehyde3-phosphate; 13DPG, 1,3-bisphospho-D-glycerate; 3 PG, 3-phosphoglycerate; 2 PG, 2-phospho-D-glycerate; PEP, phosphoenolpyruvate; PYR, pyruvate; Ac-Coa, acetyl-CoA; Lac_L, L-Lactate; FOR, formate; ACTP, acetyl phosphate; AC, acetate; ACAlD, acetaldehyde; ETOH, ethanol.

Comparison of *i*xeh674 and ec*i*xeh674 demonstrated that enzyme constraints eliminated the unrealistic linear relationship between growth rate and carbon uptake observed in the GEM. As the substrate uptake rate increased, the total enzyme amount became the primary limiting factor for growth. This constraint caused a gradual decrease in the slope of the predicted growth rate curve, reflecting the inherent upper limit to growth rate under a finite enzyme amount (Fig. 2a). Both *i*xeh674 and ec*i*xeh674 were used to predict YUAN-3's growth rate (Fig. 2b) and hydrogen yield (Fig. 2c). Comparison with experimental data showed that ec*i*xeh674 achieved substantially better agreement, avoiding the typical overestimation of maximum growth rate and hydrogen yield seen in GEM.

FBA was performed to quantify, the intrinsic trade-off between growth and hydrogen production in YUAN-3 (Fig. 2d and Supplementary Fig. S2). When growth was used as the optimization objective, rapid growth required a moderate increase in acetate flux to enhance ATP generation, with the growth rate peaking at an acetate yield of 1 mol per mol glucose (Supplementary Fig. S3). Meanwhile, the hydrogen yield, supported by elevated acetate production, remained at 2.3 mol per mol glucose. After introducing enzyme resource constraints, the simulations showed that sustaining the optimal growth rate required diverting a large fraction of the total enzyme amount toward precursor substance synthesis. Consequently, fluxes through hydrogen-producing pathways decreased markedly. The acetate flux dropped to approximately half of that predicted by the GEM, resulting in a reduced optimal hydrogen yield of 0.7 mol per mol glucose (Fig. 2e). This prediction agreed well with the experimentally observed hydrogen yields. When hydrogen production was set as the optimization objective, flux through acetate-producing pathways increased, thereby increasing ATP generation but competing for resources needed for the synthesis of biomass precursors. Simultaneously, the elevated flux through the acetate pathway increased demand for enzymatic resources, further constraining the synthesis of biomass precursors.

The experimental data were also consistent with those of ec*i*xeh674 simulations. After inoculation, the strain entered the exponential phase with optimal growth, but a hydrogen yield of only 0.3 mol per mol glucose at this stage. During the stationary growth phase, the growth rate stabilized at a lower level. In contrast, the hydrogen yield increased to 1.8 mol per mol glucose (Fig. 2f). This result suggests that, under resource-limited physiological conditions, achieving a high hydrogen yield requires a trade-off between growth rate and enzyme utilization, which can be optimized through metabolic engineering. The ec*i*xeh674 quantitatively predicted the growth rate and hydrogen yield of YUAN-3 and elucidated the intrinsic trade-off between growth and hydrogen production at the system level. This study provides a theoretical basis for future metabolic engineering strategies to enhance hydrogen yield.

### 3.3 Exploration of YUAN-3 hydrogen production mechanisms

**Fig. 3:**
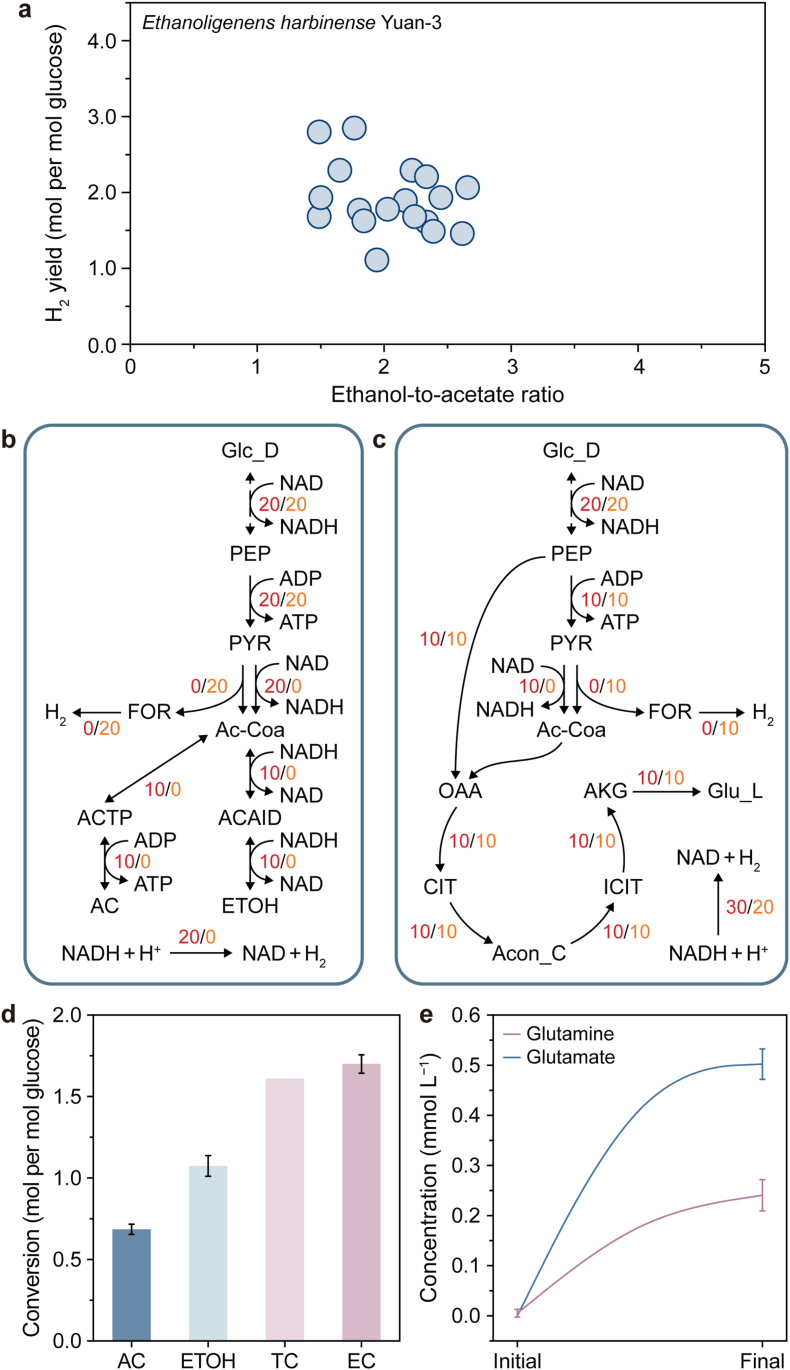
**Mechanisms of amino acid distribution in hydrogen production by YUAN-3. a**, Hydrogen yield at different ethanol-to-acetate ratios from previous studies. **b**, Metabolic fluxes of hydrogen production in the acetate and ethanol pathways. **c**, Metabolic fluxes of hydrogen production in amino acid biosynthesis pathways. Flux values are colored according to condition: red, flux through the NAD-dependent pyruvate-to-acetyl-CoA conversion, accompanied by NADH generation; orange, flux through the pyruvate formate-lyase route, producing formate and acetyl-CoA. **d**, Yields of acetate, ethanol, and hydrogen during the high hydrogen production phase. TC represents the hydrogen yield calculated based on metabolic byproducts; EC represents the observed hydrogen yield from experiments. **e**, Changes in glutamate and glutamine during the high hydrogen production phase. Error bars represent the standard deviations of parallel samples. Glc_D, D-glucose; PEP, phosphoenolpyruvate; PYR, pyruvate; Ac-Coa, acetyl-CoA; FOR, formate; ACTP, Acetyl phosphate; AC, acetate; ACAlD, acetaldehyde; ETOH, ethanol; OAA, oxaloacetate; CIT, citrate; Acon_C, cis-Aconitate; ICIT, isocitrate; AKG, 2-Oxoglutarate; Glu_L, L-Glutamate.

In previous studies, YUAN-3 primarily produced ethanol and acetate as the main metabolic byproducts, with molar ratios typically ranging from one to two. Despite this, the strain maintained a relatively high hydrogen yield (Fig. 3a). To further investigate its metabolic behavior, the ethanol-to-acetate ratio was applied as a constraint within ec*i*xeh674. FBA was then performed with maximal hydrogen production as the objective, without imposing a minimum growth constraint, predicting a maximum hydrogen yield of 3 mol of hydrogen per mol of glucose. Flux analysis indicated that the increased hydrogen yield resulted from the diversion of phosphoenolpyruvate and acetyl-CoA fluxes toward glutamate and glutamine biosynthesis (Fig. 3c). In contrast, the biosynthesis of other amino acids did not significantly contribute to the hydrogen yield. This metabolic diversion reduced flux through the ethanol pathway, lowering reduced nicotinamide adenine dinucleotide (NADH) consumption and channeling a greater proportion of NADH toward hydrogen production (Fig. 3b and Supplementary Table S3). Compared with the conventional ethanol- and butyrate-producing pathways, the theoretical maximum hydrogen yield increased by approximately 50% (Supplementary Text S7 and Supplementary Fig. S4).

Experimental validation confirmed the model predictions. During the high-efficiency hydrogen-producing phase, YUAN-3 exhibited an ethanol-to-acetate ratio of 1.6 (Fig. 3d and Supplementary Fig. S5). The hydrogen yield at this stage was 1.7 mol per mol glucose, slightly higher than the theoretical yield predicted by conventional pathways (Fig. 3d and Supplementary Fig. S6). Measurement of amino acid concentrations during hydrogen production revealed the accumulation of glutamate and glutamine (Fig. 3e). In contrast, the concentrations of other amino acids did not show significant changes (Supplementary Fig. S7). This observation corroborates the model-predicted diversion of flux toward amino acid biosynthesis. These results suggest that the biosynthesis of glutamate and glutamine serves as an alternative metabolic route for distributing both NADH and carbon flux during anaerobic biohydrogen production. Meanwhile, the redistribution of NADH and its interplay with NADPH, transhydrogenase activity, and nitrogen assimilation costs require targeted measurements at the cofactor or flux level for further mechanistic verification.

### 3.4 Genetic strategies for hydrogen enhancement

**Fig. 4:**
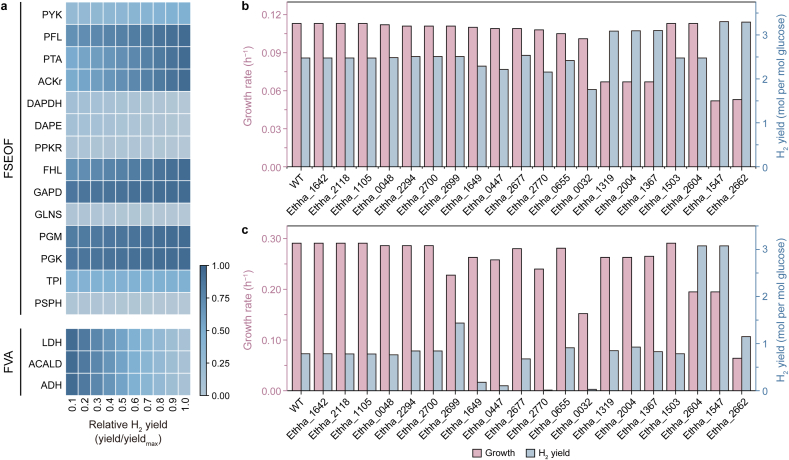
**Model-based simulation of genetic interventions for enhanced hydrogen production. a**, Flux scanning based on enforced objective flux (FSEOF) and flux variability analysis (FVA) of reaction overexpression targets for hydrogen production. The vertical axis represents reaction overexpression targets, and the horizontal axis indicates the relative hydrogen production rate. The color gradient from light blue to dark blue reflects the corresponding reaction flux from low to high. PYK, pyruvate kinase; PFL, pyruvate formate lyase; PTA, phosphotransacetylase; ACKr, acetate kinase; DAPDH, diaminopimelate dehydrogenase; DAPE, Diaminopimelate epimerase; PPKR, Polyphosphate kinase; FHL, Formate-hydrogen lyase; GAPD, Glyceraldehyde-3-phosphate dehydrogenase; GLNS, Glutamine synthetase; PGM, Phosphoglycerate mutase; PGK, Phosphoglycerate kinase; TPI, Triose-phosphate isomerase; LDH, L-lactate dehydrogenase; ACALD, Acetaldehyde dehydrogenase; ADH, ethanol: NAD ^+^ oxidoreductase. **b**–**c**, Comparison of hydrogen yield and cell growth rate following single-gene knockouts under low (**b**) and high (**c**) carbon uptake conditions. WT denotes the original model.

Within the ecGEM framework, we conducted single-gene knockout simulations to maximize the growth rate. The results showed that deletion of 20 genes led to varying degrees of growth reduction. Based on prior analyses revealing a trade-off between growth and hydrogen production, we further simulated the effects of these knockouts on both growth performance and hydrogen production. Under low-carbon uptake conditions, five gene knockouts were predicted to significantly enhance hydrogen yield (Fig. 4b). Among them, Ethha_1547 (phosphoglycerate kinase) had the greatest impact, increasing hydrogen flux by approximately 30%. Flux analysis indicated that its deletion reduced ATP synthesis, prompting the cell to increase acetate production to maintain energy balance and thereby improve hydrogen yield. These findings suggest that modulating energy generation and allocation can help rebalance growth and hydrogen production at the systems level. At high carbon uptake rates, most gene knockouts no longer improved hydrogen production due to resource limitations for enzymes. Only Ethha_2699, Ethha_2604, and Ethha_1547 continued to show a positive effect on hydrogen flux (Fig. 4c).

Additionally, flux scanning, based on the enforced objective flux algorithm, was used to identify potential overexpressed targets associated with hydrogen production. By gradually increasing hydrogen flux under constant cultivation conditions, we analyzed the corresponding response patterns of metabolic network fluxes. The results showed that certain metabolic reactions exhibited a stable linear correlation with increasing hydrogen flux (Fig. 4a). When combined with flux variability analysis, competitive metabolic branches that could divert flux away from hydrogen production were identified. These insights provide a theoretical basis for the coordinated design of overexpression and repression strategies in future studies. Although some gene knockouts significantly enhanced hydrogen yield in silico, such improvements often come at the cost of reduced growth rates, which may limit their applicability in industrial fermentation processes. Therefore, when designing metabolic engineering strategies, a careful balance must be struck between enhancing product yield and maintaining cell growth performance, aiming for optimization routes with greater engineering feasibility.

### 3.5 Industrial application potential of ecGEM in biohydrogen production

The ecGEM serves as a tool for deciphering cellular metabolic mechanisms and a quantitative platform for rational strain design and process optimization. By integrating metabolic network topology with enzyme resource constraints at the whole-cell level, ecGEM enables systematic evaluation of trade-offs between cellular metabolism and hydrogen production and the prediction of theoretical production limits. These features make ecGEM particularly valuable for guiding the development of robust, high-yield strains under industrial conditions.

In addition to its role in strain-level engineering, ecGEM also offers clear advantages for addressing challenges associated with mixed substrates and community stability in industrial biohydrogen production. Biomass pretreatment and hydrolysis often generate mixed-sugar substrates, primarily composed of D-glucose and D-xylose. However, due to carbon catabolite repression, microorganisms preferentially consume glucose, limiting the overall conversion efficiency of the sugar mixture [29]. Overcoming substrate competition and metabolic bottlenecks to achieve efficient co-utilization of all sugar components remains a major bottleneck for industrialization [30]. In this context, the ecGEM framework demonstrates strong methodological scalability. By incorporating interspecies metabolite exchange and resource competition, it can also be extended to multi-species models. Thus, enabling the simulation of metabolic division of labor and cooperative hydrogen production in mixed microbial communities. This provides a theoretical basis for optimizing mixed-culture fermentation systems in industrial settings. By integrating with system-level research on hydrogen production, storage, and utilization, ecGEM-based modeling approaches hold promise for future combination with process parameter optimization and reactor integration, thereby supporting the scale-up and industrialization of biohydrogen technologies.

## 4 Conclusion

This study constructed a GEM for the YUAN-3 strain to improve the systematic understanding of anaerobic biohydrogen production. We further refined the model by incorporating *k*_cat_ values, which enabled a more accurate simulation of the strain's metabolic behavior. The resulting ecGEM showed strong consistency with experimental data for both carbon-source utilization and growth performance. Experimental validation confirmed the metabolic trade-offs and potential regulatory mechanisms of hydrogen production elucidated by the ecGEM. Predictive analyses also identified potential gene targets for metabolic engineering of hydrogen-producing pathways and highlighted the ecGEM framework's potential industrial applicability to anaerobic biohydrogen production. Together, these results provide a foundation for the characterization and mechanistic understanding of anaerobic biohydrogen production systems and support a shift from empirical to model-guided approaches.

## CRediT authorship contribution statement

**Wei Xing:** Writing – original draft, Software, Methodology, Investigation, Conceptualization. **Jianfeng Liu:** Writing – review & editing, Methodology, Funding acquisition, Formal analysis, Data curation, Conceptualization, Visualization, Writing – original draft. **Bin Liu:** Methodology, Conceptualization. **Yanan Hou:** Methodology, Data curation, Funding acquisition. **Jia Zhang:** Validation, Investigation. **Shuang Gao:** Methodology, Investigation. **Ai-Jie Wang:** Resources, Methodology, Conceptualization. **Qianqian Yuan:** Writing – review & editing, Software, Resources, Methodology, Conceptualization. **Nan-Qi Ren:** Writing – review & editing, Resources, Project administration, Funding acquisition, Conceptualization. **Cong Huang:** Writing – review & editing, Supervision, Project administration, Funding acquisition, Conceptualization.

## Declaration of competing interest

The authors declare the following financial interests/personal relationships which may be considered as potential competing interests: Professor Nan-Qi Ren (Editor-in-Chief) and Professor Ai-Jie Wang (Executive Editor), being current editors of this journal, were entirely recused from the peer review, editorial decision-making, and manuscript handling processes.

## Data Availability

The data and the related code for the model are publicly available. All resources will be released at .https://github.com/xw685813-arch/ecixeh674.
