# Supplementary material for: Enzyme-constrained genome-scale modeling resolves growth-production trade-offs in fermentative biohydrogen production
Source: Environ Sci Ecotechnol. 2026 May 14;31:100706. doi: 10.1016/j.ese.2026.100706 (PMC13213674; doi:10.1016/j.ese.2026.100706)
Supplement: Multimedia component 1 [file mmc1.docx]

**Supporting Information**

**Enzyme-constrained genome-scale modeling resolves growth-production trade-offs in fermentative biohydrogen production**

Wei Xing^a,1^, Jianfeng Liu^b,1^, Bin Liu^b^, Yanan Hou^a,c^, Jia Zhang^a^, Shuang Gao^a^, Ai-Jie Wang^b,d^, Qianqian Yuan^a,*^, Nan-Qi Ren^a,d,*^, Cong Huang^a,*^

a National Technology Innovation Center of Synthetic Biology, Tianjin Institute of Industrial Biotechnology, Chinese Academy of Sciences, Tianjin, 300308, China

b Key Laboratory of Environmental Biotechnology, Research Center for Eco-Environmental Sciences, Chinese Academy of Sciences, Beijing 100085, China

c Tianjin Key Laboratory of Aquatic Science and Technology, School of Environmental and Municipal Engineering, Tianjin Chengjian University, Tianjin, 300384, China

d State Key Laboratory of Urban Water Resource and Environment, Harbin Institute of Technology (Shenzhen), Shenzhen, 518055, China

**Summary Information**Number of pages: 9

Texts: S1–S7
Figures: S1–S7
Table: S1

**Supplementary Methods**

**Texts S1 Gas collection**

Gas production was quantified using a gas-tight syringe method under sealed conditions. Gas samples were collected from the headspace of anaerobic serum bottles using a 50 mL gas-tight syringe. The syringe needle was inserted through the butyl rubber stopper and held in place until pressure equilibrium between the syringe and the bottle headspace was achieved. The syringe was then withdrawn, and the extracted gas volume was recorded. Hydrogen and carbon dioxide concentrations in the collected gas were determined by gas chromatography (GC). The total amount of each gas was calculated from the measured gas concentration and the combined gas volume, consisting of the syringe volume and the bottle headspace volume.

To calculate net gas production, the amounts of hydrogen and carbon dioxide remaining in the bottle headspace after the previous sampling were subtracted from the total gas content measured at the current sampling time. This approach corrects for residual headspace gases between successive sampling events and allows accurate estimation of cumulative gas production. Nitrogen flushing was performed only prior to sterilization to establish anaerobic conditions, and no further nitrogen replacement was applied during fermentation. All bottles remained sealed throughout incubation. Gas yields were calculated assuming ideal gas behavior at constant incubation temperature and atmospheric pressure. Because gas sampling was conducted after pressure equilibration, no additional pressure correction was applied.

**Texts S2 GEM preprocessing**

Before constructing the enzyme-constrained model, it is essential to preprocess the genome-scale metabolic network model to ensure that enzyme parameters can be correctly matched to each reaction. Firstly, all reversible reactions are split into two unidirectional irreversible reactions, assigning enzyme catalytic parameters for both the forward and reverse directions. This approach prevents a single reaction from masking the differences in enzyme efficiency between the forward and reverse pathways. Secondly, reactions catalyzed by multiple isoenzymes are separated by breaking down the original reaction into several independent reactions, each corresponding to one isoenzyme and assigned specific enzyme parameters. Through these two steps, each reaction in the model has a clear direction and unique enzymatic parameters.

**Texts S3 Acquisition of Uniprot IDs**

The protein coding sites in the genome-scale metabolic network model for YUAN-3, which were annotated using RAST, need to be converted into Uniprot ID format to facilitate the retrieval of enzyme parameter-related information. The protein information for this strain was downloaded from the Uniprot website, and by aligning the protein sequences, gene IDs in the model were converted to Uniprot IDs.

**Texts S4 Acquisition of subunit number**

The subunit number of an enzyme refers to the number of subunits (polypeptide chains) that make up the enzyme molecule. It not only affects the enzyme’s spatial conformation but also influences its functional characteristics and catalytic mechanism. The subunit number is directly related to the enzyme’s molecular weight, which is an essential parameter in enzyme-constrained models. To obtain comprehensive and accurate information on enzyme subunit numbers, they were retrieved based on UniProt IDs using ecmpy.

**Texts S5 Acquisition of enzyme mass fraction**

The protein content of YUAN-3 cells was experimentally determined, and the intracellular protein concentration per unit cell dry weight was set to 0.45 g/g DW. To calculate the enzyme mass fraction (f, the proportion of enzymes relative to the total protein) of the YUAN-3 strain, RNA-seq data of the strain were retrieved from the NCBI database. Based on RNA-seq alignment and related analyses, the relative abundance of each enzyme was determined and subsequently converted into the f within the total protein.

**Texts S6 Acquisition of Turnover numbers**

The acquisition of *k_cat_* was carried out through two approaches: database retrieval and machine learning prediction. First, enzyme parameter data of the strain were obtained from databases using AutoPACMEN. Examination of the retrieved data showed that most of the parameters were derived from other species. Therefore, this study employed DLkcat to predict enzyme parameter information. To construct the input features for the model, the SMILES representations of all metabolites in the model were automatically obtained using the ModelSEED API, while the amino acid sequences of the corresponding enzymes were retrieved from UniProt. The metabolite SMILES representations and enzyme sequences were then input into the DLKcat model to predict enzyme parameters for the target reactions. Using this approach, *k_cat_* was successfully matched for 1,006 reactions.

**Texts S7 Stoichiometric equations for scenario-specific theoretical H_2_ yields**

Hydrogen production under different metabolic substrates was simulated using a constraint-based model. During the simulations, only the types of metabolic products were constrained. For instance, in the acetate-type metabolism, acetate was set as the sole product; in the butyrate-type, butyrate was set accordingly. For ethanol-type metabolism, both ethanol and acetate were defined as products with a fixed ratio of 1:1. In amino acid metabolism, the ethanol-to-acetate production ratio was also constrained to 1:1. FBA was used to simulate the optimal solution in each case, without applying any constraint on the minimum biomass growth rate. The stoichiometric equations corresponding to each metabolic type are shown below.

Acetate pathway:

C_6_H_12_O_6_+2H_2_O → 2CH_3_COOH+2CO_2_+4H_2_ (1)

Butyrate pathway:

C_6_H_12_O_6_+2H_2_O → CH_3_CH_2_CH_2_COOH+2CO_2_+2H_2_ (2)

Ethanol pathway:

C_6_H_12_O_6_+H_2_O → CH_3_COOH+CH_3_CH_2_OH+2CO_2_+2H_2_ (3)

Glutamate pathway:

C_6_H_12_O_6_+NH_3_ → C_5_H_9_NO_4_+CO_2_+3H_2_ (4)

Glutamine pathway:

C_6_H_12_O_6_+2NH_3_ → C_5_H_10_N_2_O_3_+CO_2_+H_2_O+3H_2_ (5)

After applying a fixed acetate-to-ethanol ratio constraint (without imposing a minimum biomass growth rate), the model predicted that the maximum hydrogen yield via the amino acid metabolic pathway increased by approximately 50% compared to the ethanol pathway. When a minimum biomass growth constraint was introduced, this improvement in hydrogen yield was somewhat reduced.

**Supplementary Figures**

**
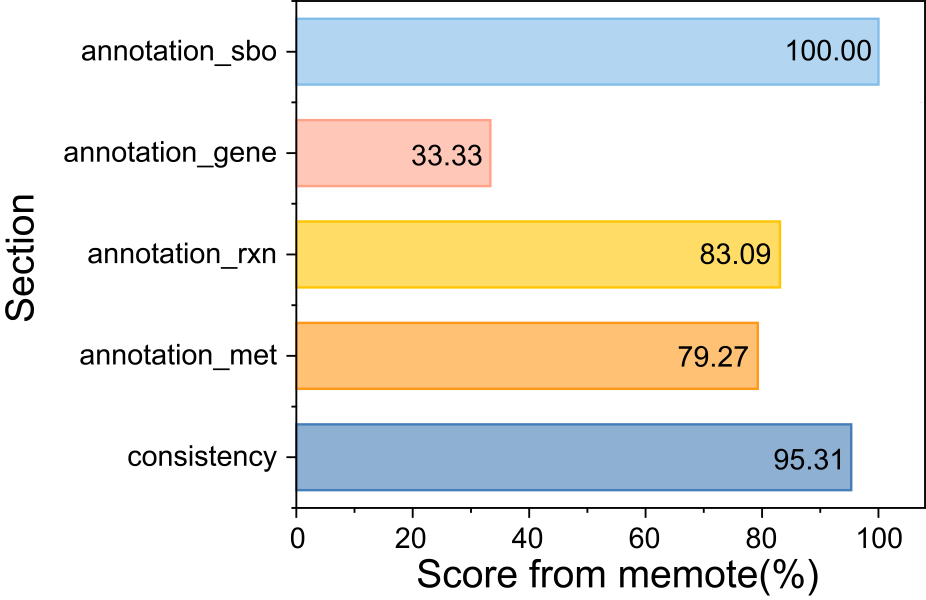
**

**Fig. S1.** Evaluation of the GEM of *Ethanoligenens harbinense* YUAN-3 using MEMOTE.


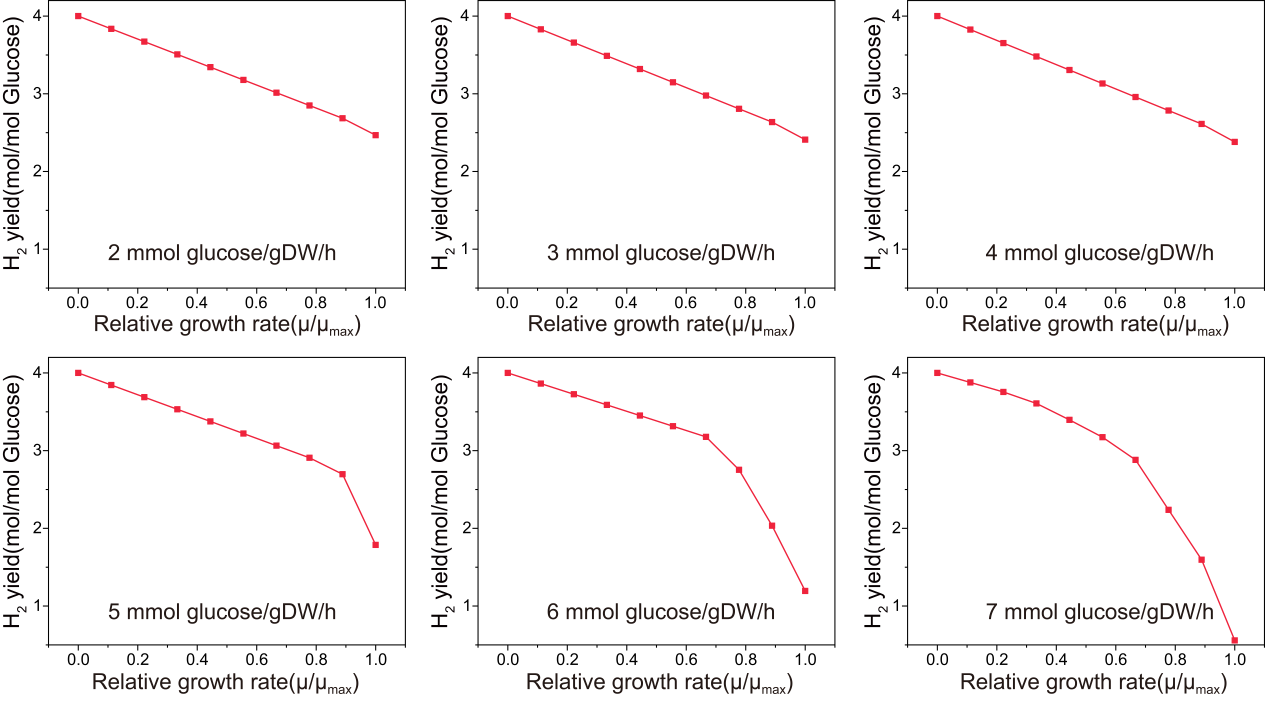


**Fig. S2.** The trade-off between hydrogen yield and growth rate under varying substrate uptake rates.


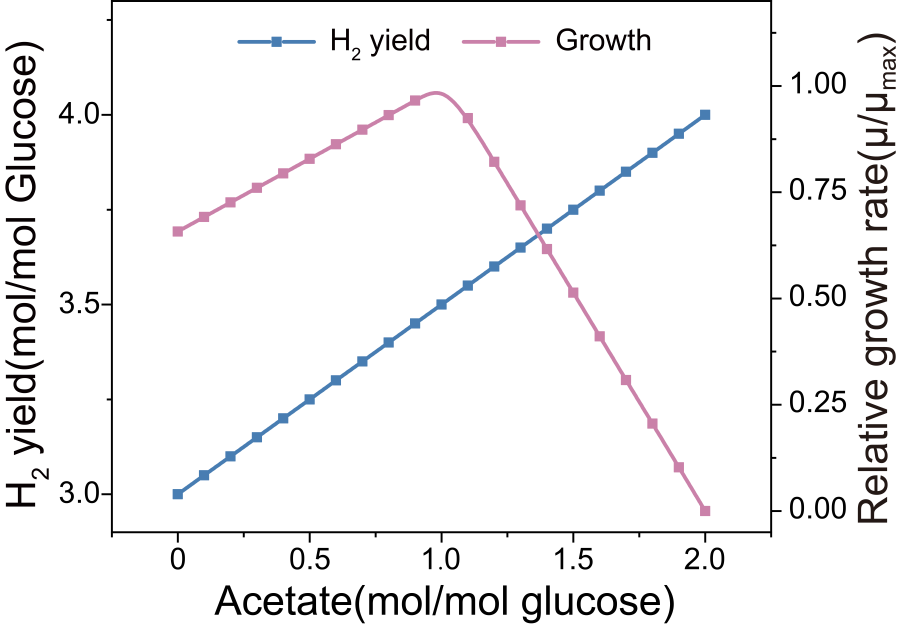


**Fig. S3.** Effect of acetate yield on hydrogen yield and growth rate.

**
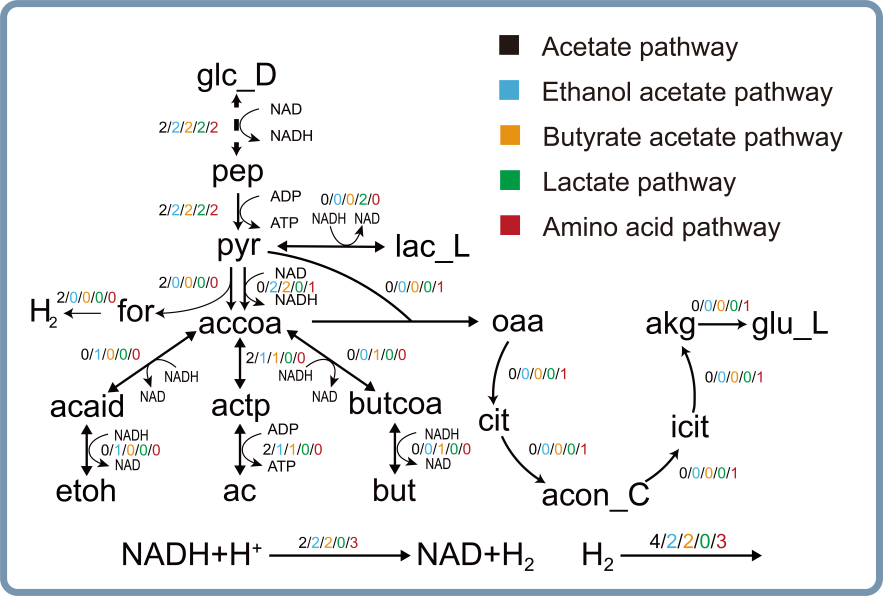
**

**Fig. S4.** Optimal hydrogen production simulated by the ec*i*xeh674 model under different metabolic pathways. Black, blue, orange, green, and red represent the acetate, ethanol, butyrate, lactate, and amino acid pathways, respectively.


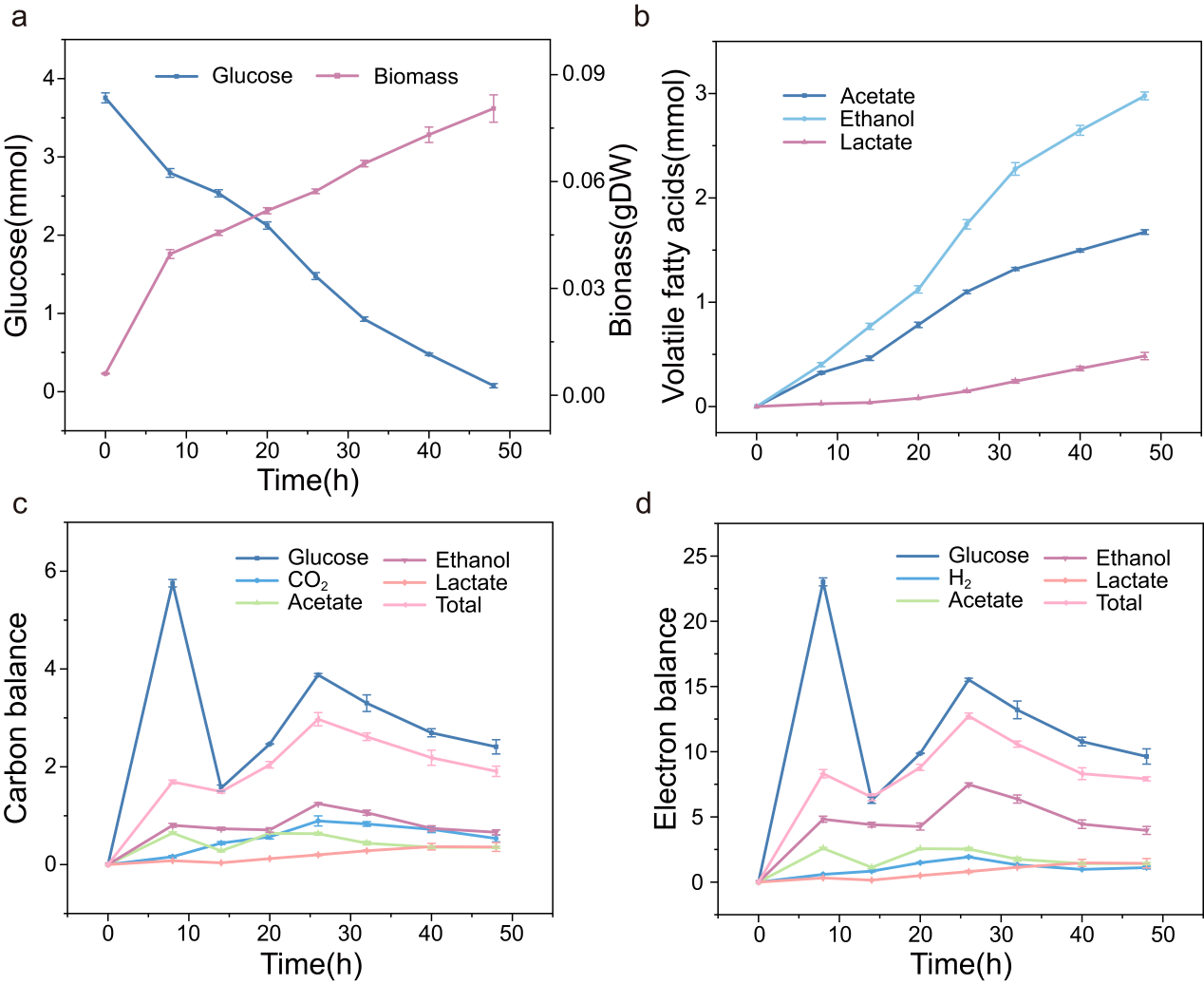


**Fig. S5.** (a) Time profiles of glucose consumption and biomass accumulation. (b) Time profiles of major soluble metabolites. (a) Carbon balance closure at each sampling time point. (b) Electron balance closure at each sampling time point.

**
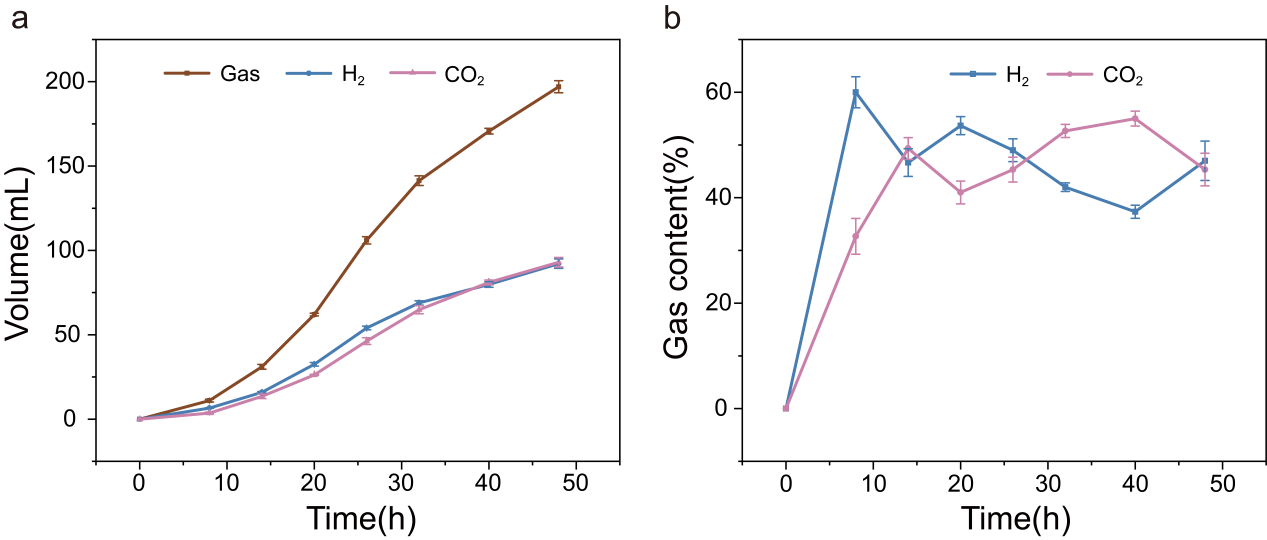
**

**Fig. S6.** (a) Temporal variation of gas volume. (b) Temporal variation of gas composition.


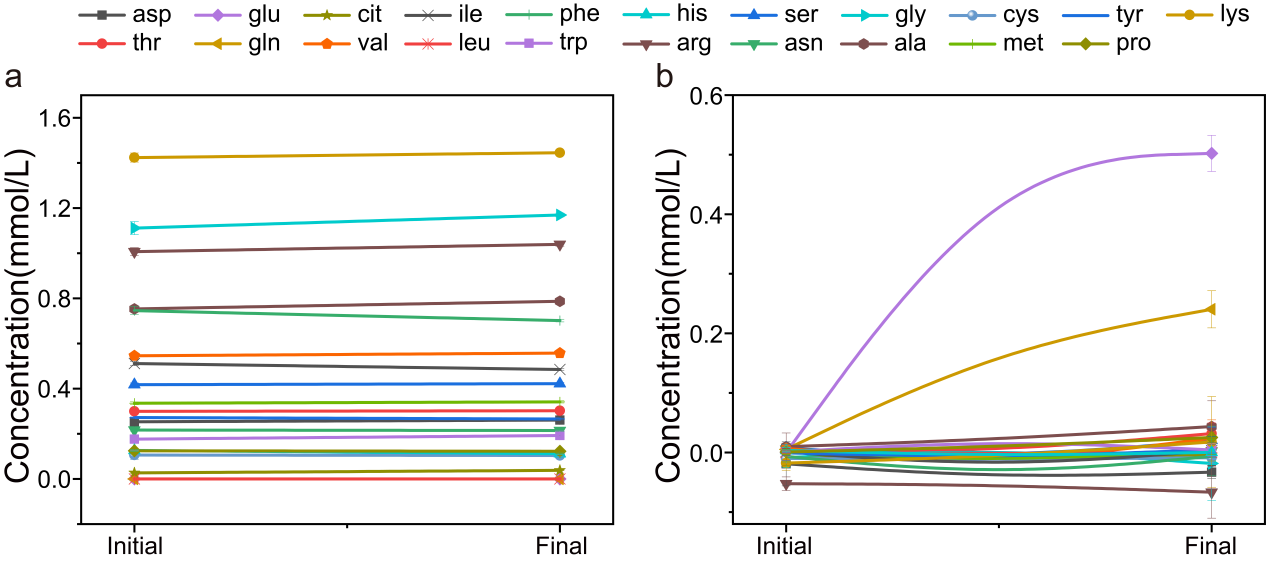


**Fig. S7.** Variation in amino acid concentrations. (a) Changes in amino acid concentrations in the culture medium. (b) Changes in amino acid concentrations during the high hydrogen production phase.

**Supplementary Table**

**Table. S1.** Newly added GPR associations

| Gene_ID | Reaction_ID | Gene_ID | Reaction_ID |
| --- | --- | --- | --- |
| Ethha_0326 | rxn16108_c0 | Ethha_0683 | rxn05039_c0 |
| Ethha_2056 | rxn13645_c0 | Ethha_0704 | rxn05039_c0 |
| Ethha_1996 | rxn13299_c0 | Ethha_0433 | rxn05039_c0 |
| Ethha_2367 | rxn11946_c0 | Ethha_0422 | rxn05039_c0 |
| Ethha_0121 | rxn11946_c0 | Ethha_0127 | rxn03397_c0 |
| Ethha_1643 | rxn09310_c0 | Ethha_0731 | rxn03167_c0 |
| Ethha_1340 | rxn05039_c0 | Ethha_2157 | rxn02304_c0 |
| Ethha_2031 | rxn05039_c0 | Ethha_0620 | rxn01258_c0 |
| Ethha_0813 | rxn05039_c0 | Ethha_1387 | rxn01208_c0 |
| Ethha_1079 | rxn05039_c0 | Ethha_1754 | rxn00379_c0 |
| Ethha_0564 | rxn05039_c0 | Ethha_0819 | rxn00379_c0 |
| Ethha_0837 | rxn05039_c0 | Ethha_1823 | rxn00062_c0 |
| Ethha_0678 | rxn08088_c0; rxn08086_c0 | Ethha_2432 | rxn06865_c0; rxn06848_c0 |
| Ethha_2414 | rxn08018_c0; rxn08016_c0 | Ethha_1091 | rxn06865_c0; rxn06848_c0 |
| Ethha_2635 | rxn06865_c0; rxn06848_c0 | Ethha_2348 | rxn06865_c0; rxn06848_c0 |
| Ethha_0810 | rxn06865_c0; rxn06848_c0 | Ethha_1088 | rxn06865_c0; rxn06848_c0 |
| Ethha_1371 | rxn06865_c0; rxn06848_c0 | Ethha_1401 | rxn06865_c0; rxn06848_c0 |
| Ethha_2089 | rxn06865_c0; rxn06848_c0 | Ethha_1163 | rxn06865_c0; rxn06848_c0 |
| Ethha_2127 | rxn06865_c0; rxn06848_c0 | Ethha_1155 | rxn06865_c0; rxn06848_c0 |
| Ethha_1472 | rxn06865_c0; rxn06848_c0 | Ethha_0462 | rxn05582_c0; rxn05496_c0 |
| Ethha_1519 | rxn06865_c0; rxn06848_c0 | Ethha_0290 | rxn08891_c0 |
| Ethha_1298 | rxn09211_c0; rxn09210_c0; rxn09208_c0 | Ethha_0315 | rxn08551_c0; rxn06865_c0; rxn06848_c0 |
